# Supplementary material for: Dietary Patterns of Patients with Prediabetes and Type 2 Diabetes
Source: Metabolites. 2023 Apr 7;13(4):532. doi: 10.3390/metabo13040532 (PMC10142065; doi:10.3390/metabo13040532)
Supplement: Supplementary file 1 [file metabolites-13-00532-s001.zip › Table S1.pdf]

Table S1. Food grouping used in the dietary pattern assessment

Food groups	Food
Meat and fish	beef: roast, steak, mince, stew or casserole, beefburgers, pork: roast, chops, stew or slices, lamb: roast, chops or stew, chicken or other poultry, bacon, ham, corned beef, spam, luncheon meats, sausages, savoury pies, liver, liver paté, liver sausage, fried fish in batter, as in fish and chips, fish fingers, fish cakes, other white fish, fresh or frozen, oily fish, fresh or canned, shellfish, fish roe, taramasalata
Bread and savoury biscuits	white bread and rolls, brown bread and rolls, wholemeal bread and rolls, cream crackers, cheese biscuits, crispbread
Cereals	porridge, Readybrek, breakfast cereal such as cornflakes, muesli etc.
Potatoes, rice and pasta	boiled, mashed, instant or jacket potatoes, chips, roast potatoes, potato salad, white rice, brown rice, white or green pasta, wholemeal pasta, lasagna, moussaka, pizza
Diary products and fats	single or sour cream, double or clotted cream, low fat yogurt, fromage frais, full fat or Greek yogurt, dairy desserts, cheese, cottage cheese, low fat soft cheese, eggs as boiled, fried, scrambled, quiche, low calorie, low fat salad cream, salad cream, mayonnaise, french dressing, other salad dressing, butter, block or hard margarine, polyunsaturated margarine, soft margarines, including olive oil based and dairy spreads, low fat spreads (less than 60% fat), very low fat spread (less than 30% fat)
Sweets and snacks	sweet biscuits, chocolate, sweet biscuits, plain, cakes home baked, cakes ready-made, buns, pastries homemade, buns, pastries ready-made, fruit pies, tarts, crumbles, home baked, fruit pies, tarts, crumbles, ready-made, sponge puddings, home baked, sponge puddings, readymade, milk puddings, ice cream, choc ices, chocolates, single or squares, chocolate snack bars, sweets, toffees, mints, sugar added to tea, coffee, cereal, crisps or other packet snacks, peanuts or other nuts
Soups, sauces, and spreads	vegetable soups, meat soups, sauces, tomato ketchup, pickles, chutney, marmite, bovril, jam, marmalade, honey, peanut butter
Drinks	tea, coffee, instant or ground, coffee, decaffeinated, coffee whitener, cocoa, hot chocolate, horlicks, ovaltine, wine, beer, lager or cider, port, sherry, vermouth, liqueurs, spirits, low calorie or diet fizzy soft drinks, fizzy soft drinks, pure fruit juice (100%), fruit squash or cordial
Fruit	apples, pears, oranges, satsumas, mandarins, grapefruit, bananas, grapes, melon, peaches, plums, apricots, strawberries, raspberries, kiwi fruit, tinned fruit, dried fruit
Vegetables	carrots, spinach, broccoli, spring greens, kale, brussels sprouts, cabbage, peas, green beans, broad beans, runner beans, marrow, courgettes, cauliflower, parsnips, turnips, swedes, leeks, onions, garlic, mushrooms, sweet peppers, beansprouts, green salad, lettuce, cucumber, celery, watercress, tomatoes, sweetcorn, beetroot, coleslaw, avocado, baked beans, dried lentils, beans, peas, tofu , soya meat, TVP, vegeburger
