# Supplementary material for: Dietary Patterns of Patients with Prediabetes and Type 2 Diabetes
Source: Metabolites. 2023 Apr 7;13(4):532. doi: 10.3390/metabo13040532 (PMC10142065; doi:10.3390/metabo13040532)
Supplement: Supplementary file 1 [file metabolites-13-00532-s001.zip › Table S2.pdf]

Table S2. Food frequency of patients in the two groups

Foods and Amounts	Prediabetic	T2DM	p value
Meat and fish (medium serving)			
Beef: roast, steak, mince, stew or casserole	2.1 ± 1.0	2.1 ± 1.1	0.791
Beefburgers	1.2 ± 0.4[a]	1.1 ± 0.3[b]	**0.002**
Pork: roast, chops, stew or slices	2.6 ± 1.1	2.6 ± 1.2	0.978
Lamb: roast, chops or stew	1.2 ± 0.6	1.2 ± 0.5	0.718
Chicken or other poultry eg. turkey	4.0 ± 1.0	4.1 ± 1.0	0.245
Bacon	1.3 ± 0.7	1.3 ± 0.7	0.520
Ham	1.9 ± 1.1	1.8 ± 1.0	0.133
Corned beef, Spam, luncheon meats	1.0 ± 0.9	1.0 ± 0.9	0.668
Sausages	2.0 ± 1.1 [a]	1.7 ± 1.0 [b]	**0.000**
Savoury pies, eg. meat pie, pork pie, pasties, steak & kidney pie, sausage rolls	1.3 ± 0.7 [a]	1.1 ± 0.3 [b]	**0.002**
Liver, liver paté, liver sausage	1.3 ± 0.7	1.2 ± 0.3	0.021
Fried fish in batter, as in fish and chips	1.4 ± 0.7 [a]	1.5 ± 0.8 [b]	**0.031**
Fish fingers, fish cakes	1.1 ± 0.4	1.1 ± 0.4	0.600
Other white fish, fresh or frozen, eg. cod, haddock, plaice, sole, halibut	2.2 ± 0.9	2.2 ± 1.0	0.935
Oily fish, fresh or canned, eg. mackerel, kippers, tuna, salmon, sardines, herring	1.0 ± 0.6	1.0 ± 0.1	0.318
Shellfish, eg. crab, prawns, mussels	1.1 ± 0.4	1.1 ± 0.3	0.140
Fish roe, taramasalata	1.3 ±0.6	1.3 ± 0.6	0.929
Bread and savoury biscuits (one slice or biscuit)			
White bread and rolls	4.7 ± 2.8	4.4 ± 3.0	0.300
Brown bread and rolls	3.4 ± 2.6	3.4 ± 2.8	0.699
Wholemeal bread and rolls	3.5 ± 2.6	3.2 ± 2.7	0.116
Cream crackers, cheese biscuits	1.1 ± 0.5	1.1 ± 0.4	0.083
Crispbread	1.2 ± 0.6	1.1 ± 0.6	0.361
Cereals (one bowl)			
Porridge, Readybrek	1.3 ± 0.8	1.2 ± 0.7	0.081
Breakfast cereal such as cornflakes, muesli etc.	1.6 ± 1.1 [a]	1.4 ± 0.9 [b]	**0.020**
Potatoes, rice and pasta (medium serving)			
Boiled, mashed, instant or jacket potatoes	3.4 ± 1.1	3.3 ± 1.0	0.382
Chips	2.2 ± 1.0 [a]	2.0 ± 1.0 [b]	**0.009**
Roast potatoes	2.0 ± 1.0	1.8 ± 1.0	0.076
Potato salad	1.0 ± 0.2	1.0 ± 0.1	0.318
White rice	2.6 ± 1.0	2.6 ± 0.9	0.590
Brown rice	1.2 ± 0.6	1.1 ± 0.5	0.217
White or green pasta, eg. Spaghetti, macaroni, noodles	2.4 ± 0.9	2.4 ± 0.9	0.483
Wholemeal pasta	1.2 ± 0.6	1.1 ± 0.5	0.325
Lasagne, moussaka	1.3 ± 0.5 [a]	1.1 ± 0.4 [b]	**0.000**
Pizza	1.6 ± 0.7 [a]	1.4 ± 0.6 [b]	**0.000**
Single or sour cream (tablespoon)	1.9 ± 1.3	1.9 ± 1.2	0.690
Double or clotted cream (tablespoon)	2.3 ± 1.3	2.2 ± 1.2	0.232
Low fat yogurt, fromage frais (125g carton)	2.4 ± 1.6	2.3 ± 1.6	0.246
Full fat or Greek yogurt (125g carton)	2.6 ± 1.6	2.6 ± 1.7	0.821
Dairy desserts (125g carton)	1.1 ± 0.5	1.1 ± 0.4	0.348

Cheese, eg. Cheddar, Brie, Edam (medium serving)	1.5 ± 1.0	1.5 ± 0.9	0.713
Cottage cheese, low fat soft cheese (medium serving)	3.2 ± 1.1	3.2 ± 1.2	0.713
Eggs as boiled, fried, scrambled, etc. (one)	3.7 ± 1.1	3.8 ± 1.0	0.641
Quiche (medium serving)	1.0 ± 0.2	1.1 ± 0.4	0.130
Low calorie, low fat salad cream(tablespoon)	1.1 ± 0.3	1.1 ± 0.3	0.404
Salad cream, mayonnaise (tablespoon)	1.2 ± 0.5 [a]	1.1 ± 0.4 [b]	**0.026**
French dressing (tablespoon)	1.1 ± 0.5	1.2 ± 0.7	0.763
Other salad dressing (tablespoon)	3.6 ± 1.9	3.4 ± 2.0	0.085
The following on bread or vegetables			
Butter (teaspoon)	2.2 ± 1.3	2.3 ± 1.3	0.569
Block or hard margarine (teaspoon)	1.3 ± 0.8	1.4 ± 0.9	0.824
Polyunsaturated margarine (teaspoon)	1.2 ± 0.6	1.2 ± 0.6	0.851
Soft margarines, including olive oil based and dairy spreads (teaspoon)	1.3 ± 0.8	1.2 ± 0.7	0.051
Low fat spreads (less than 60% fat) (teaspoon)	1.1 ± 0.5	1.1 ± 0.3	0.127
Very low fat spread (less than 30% fat) (teaspoon)	1.1 ± 0.3	1.0 ± 0.2	0.258
Sweets and snacks (medium serving)			
Sweet biscuits, chocolate , eg. digestive (one)	1.6 ± 1.0	1.5 ± 0.9	0.161
Sweet biscuits, plain, eg. Nice, ginger (one)	1.9 ± 1.2	1.8 ± 1.1	0.492
Cakes eg. fruit, sponge, home baked	1.3 ± 0.6 [a]	1.2 ± 0.4 [b]	**0.018**
Cakes eg. fruit, sponge, ready made	1.2 ± 0.5	1.1 ± 0.4	0.232
Buns, pastries eg. scones, flapjacks, home baked	1.1 ± 0.7	1.1 ±0.4	0.114
Buns, pastries eg. croissants, doughnuts, ready made	1.1 ± 0.3	1.1 ± 0.5	0.076
Fruit pies, tarts, crumbles, home baked	1.7 ± 0.7 [a]	1.6 ± 0.7 [b]	**0.036**
Fruit pies, tarts, crumbles, ready made	1.2 ± 0.5	1.1 ± 0.4	0.155
Sponge puddings, home baked	1.3 ± 0.7	1.2 ± 0.5	0.178
Sponge puddings, ready made	1.1 ± 0.3	1.0 ± 0.2	0.129
Milk puddings, eg. rice, custard, trifle	1.6 ± 0.8 [a]	1.4 ± 0.8 [b]	**0.016**
Ice cream, choc ices	2.3 ± 1.1	2.1 ± 1.1	0.172
Chocolates, single or squares	2.2 ± 1.2	2.2 ±1.3	0622
Chocolate snack bars	1.3 ± 0.7 [a]	1.1 ± 0.5 [b]	**0.015**
Sweets, toffees, mints	1.6 ± 1.1	1.5 ± 1.0	0.178
Sugar added to tea, coffee, cereal (teaspoon)	2.9 ± 2.2 [a]	2.1 ± 1.9 [b]	**0.000**
Crisps or other packet snacks	1.5 ± 0.9 [a]	1.3 ± 0.7 [b]	**0.001**
Peanuts or other nuts	2.2 ± 1.2	2.1 ± 1.2	0.140
Soups, sauces, and spreads			
Vegetable soups (bowl)	4.1 ± 1.2	4.1 ± 1.1	0.937
Meat soups (bowl)	3.8 ± 1.1	3.9 ± 1.0	0.087
Sauces, eg. white sauce, cheese sauce, gravy (tablespoon)	1.3 ± 0.7 [a]	1.2 ± 0.6 [b]	**0.011**
Tomato ketchup (tablespoon)	2.7 ± 1.7 [a]	2.2 ± 1.4 [b]	**0.000**
Pickles, chutney (tablespoon)	2.6 ± 1.3	2.5 ± 1.3	0.799
Marmite, Bovril (teaspoon)	1.1 ± 0.3	1.0 ± 0.3	0.377
Jam, marmalade, honey (teaspoon)	2.0 ± 1.3	2.0 ± 1.3	0.557
Peanut butter (teaspoon)	1.1 ± 0.5	1.1 ± 0.5	0.904
Drinks			
Tea (cup)	4.3 ± 1.9	4.2 ± 2.0	0.545
Coffee, instant or ground (cup)	4.8 ± 2.1	4.9 ± 2.1	0.837
Coffee, decaffeinated (cup)	1.2 ± 0.9	1.1 ± 0.7	0.117

Coffee whitener, eg. Coffee-mate (teaspoon)	1.6 ± 1.5	1.4 ± 1.3	0.055
Cocoa, hot chocolate (cup)	1.2 ± 0.7	1.1 ± 0.7	0.448
Horlicks, Ovaltine (cup)	1.0 ± 0.3	1.0 ± 0.3	0.572
Wine (glass)	1.5 ± 1.0 [a]	1.7 ± 1.2 [b]	**0.017**
Beer, lager or cider (half pint)	1.3 ± 0.8	1.3 ± 0.7	0.297
Port, sherry, vermouth, liqueurs (glass)	1.0 ± 03	1.0 ± 0.3	0.988
Spirits, eg. gin, brandy, whisky, vodka (single)	1.4 ± 0.9	1.3 ± 0.8	0.385
Low calorie or diet fizzy soft drinks (glass)	1.2 ± 0.5	1.2 ± 0.6	0.597
Fizzy soft drinks, eg. Coca cola, lemonade (glass)	1.5 ± 0.9	1.3 ± 0.8	0.053
Pure fruit juice (100%) eg. orange, apple juice (glass)	1.5 ± 1.1	1.4 ± 0.9	0.073
Fruit squash or cordial (glass)	1.1 ± 0.6	1.1 ± 0.5	0.145
Fruit			
Apples (1 fruit)	4.6 ± 1.5 [a]	4.9 ± 1.4 [b]	**0.002**
Pears (1 fruit)	2.3 ± 1.2	2.3 ± 1.3	0.483
Oranges, satsumas, mandarins (1 fruit)	3.0 ± 1.2	3.1 ± 1.4	0.214
Grapefruit (half)	2.5 ± 1.2	2.7 ±1.4	0,052
Bananas (1 fruit)	2.5 ± 1.2	2.6 ± 1.4	0.510
Grapes (medium serving)	2.3 ± 1.1 [a]	2.6 ± 1.4 [b]	**0.002**
Melon (1 slice)	2.6 ± 1.4	2.7 ± 1.4	0.371
Peaches, plums, apricots (1 fruit)	3.3 ± 1.3 [a]	3.1 ± 1.3 [b]	**0.020**
Strawberries, raspberries, kiwi fruit (medium serving)	3.1 ± 1.3	3.1 ± 1.4	0.754
Tinned fruit (medium serving)	1.7 ± 0.9 [a]	1.5 ± 0.9 [b]	**0.031**
Dried fruit, eg. raisins, prunes (medium serving)	1.4 ± 0.9	1.3 ± 0.7	0.277
Vegetables fresh, frozen or tinned (medium serving)			
Carrots	5.0 ± 1.3	5.2 ± 1.1	0.125
Spinach	2.0 ± 1.3	1.9 ± 1.1	0.334
Broccoli, spring greens, kale	1.7 ± 1.0	1.7 ± 1.0	0.511
Brussels sprouts	2.5 ± 0.9	2.5 ± 1.0	0.921
Cabbage	2.9 ± 0.9	2.8 ± 1.1	0.141
Peas	2.2 ± 0.9	2.1 ± 0.9	0.711
Green beans, broad beans, runner beans	2.4 ± 0.8	2.3 ± 1.0	0.647
Marrow, courgettes	2.1 ± 1.0	2.1 ± 0.9	0.652
Cauliflower	1.9 ± 0.9	2.0 ± 1.0	0.374
Parsnips, turnips, swedes	2.4 ± 1.3 [a]	3.1 ± 1.7 [b]	**0.031**
Leeks	1.5 ± 1.0 [a]	1.3 ± 0.7 [b]	**0.002**
Onions	4.9 ± 1.2 [a]	5.1 ± 1.0 [b]	**0.003**
Garlic	3.6 ± 1.4	3.7 ± 1.4	0.169
Mushrooms	2.6 ± 0.9	2.6 ± 1.0	0.973
Sweet peppers	2.9 ± 1.2 [a]	3.1 ± 1.2 [b]	**0.031**
Beansprouts	2.5 ± 0.9	2.5 ± 1.0	0.610
Green salad, lettuce, cucumber, celery	2.9 ± 1.3 [a]	3.2 ± 1.5 [b]	**0.005**
Watercress	1.3 ± 1.0	1.2 ± 0.7	0.104
Tomatoes	4.1 ± 1.4	4.3 ± 1.4	0.079
Sweetcorn	1.5 ± 1.0 [a]	1.4 ± 0.7 [b]	**0.040**
Beetroot	2.1 ± 0.8	2.1 ± 0.9	0,282
Coleslaw	2.8 ± 0.9	2.8 ± 1.1	0.654
Avocado	1.4 ± 0.9	1.3 ± 0.8	0.141
Baked beans	1.8 ± 0.6 [a]	1.7 ± 0.6 [b]	**0.011**
Dried lentils, beans, peas	1.7 ± 0.7	1.8 ± 0.9	0,070

Tofu , soya meat, TVP, Vegeburger	1.3 ± 0.5 [a]	1.2 ± 0.4 [b]	**0.002**

1 (never or less than once/month), 2 (1-3 per month), 3 (once a week), 4 (2-4 per week), 5 (5-6 per week), 6 (once a day), 7 (2-3 per day), 8 (4-5 per day), 9 (6+ per day). Variables are presented as mean ± SD.
