# Supplementary material for: Dietary Patterns of Patients with Prediabetes and Type 2 Diabetes
Source: Metabolites. 2023 Apr 7;13(4):532. doi: 10.3390/metabo13040532 (PMC10142065; doi:10.3390/metabo13040532)
Supplement: Supplementary file 1 [file metabolites-13-00532-s001.zip › Table S3.pdf]

Table S3. Correlation analyses between dietary intake and anthropometric measurements

	Prediabetic						T2DM					
	Weight (kg)		WC (cm)		BF (%)		Weight (kg)		WC (cm)		BF (%)	
	**r**	**p**	**r**	**p**	**r**	**p**	**r**	**p**	**r**	**p**	**r**	**p**
Meat and meat products (g)	0.142	0.001	0.127	0.002	0.017	0.783	0.052	0.209	0.055	0.180	0.046	0.270
Cereals and cereal products (g)	-0.006	0.922	-0.018	0.775	-0.127	0.003	-0.037	0.372	-0.063	0.128	-0.147	0.018
Fruit (g)	0.102	0.099	0.038	0.535	0.053	0.393	0.134	0.030	0.057	0.171	0.043	0.306

WC, waist circumference; BF, body fat.
