# Supplementary material for: Dietary Patterns of Patients with Prediabetes and Type 2 Diabetes
Source: Metabolites. 2023 Apr 7;13(4):532. doi: 10.3390/metabo13040532 (PMC10142065; doi:10.3390/metabo13040532)
Supplement: Supplementary file 1 [file metabolites-13-00532-s001.zip › Table S4.pdf]

Table S4. Correlation analyses between dietary intake and biomarkers of glycemic control

	**Prediabetic**						**T2DM**					
	FPG (mg/dL)		HbA1c (%)		SI (µIU/mL)		FPG (mg/dL)		HbA1c (%)		SI (µIU/mL)	
	**r**	**p**	**r**	**p**	**r**	**p**	**r**	**p**	**r**	**p**	**r**	**p**
Potatoes (g)	0.151	0.000	0.040	0.520	0.013	0.839	0.061	0.274	0.066	0.237	0.073	0.079
Soups & sauces (g)	0.086	0.048	0.029	0.644	0.115	0.062	0.079	0.066	0.033	0.562	0.102	0.068
Meat and meat products (g)	0.099	0.111	0.119	0.042	0.034	0.591	0.002	0.964	0.043	0.300	0.001	0.975
Milk and milk products (g)	0.073	0.240	0.076	0.237	0.237	0.000	0.190	0.002	0.032	0.463	0.006	0.916
Nuts and seeds (g)	-0.034	0.588	-0.016	0.039	0.115	0.062	-0.026	0.536	-0.031	0.465	0.062	0.267
Fruit (g)	0.105	0.089	0.097	0.118	0.068	0.287	0.184	0.003	0.057	0.310	0.023	0.579
Fats and oils (g)	-0.053	0.395	-0.097	0.116	-0.039	0.547	-0.012	0.764	-0.167	0.007	-0.011	0.791

FPG, fasting plasma glucose; HbA1c, glycated hemoglobin; SI, serum insulin.
