# Supplementary material for: Dietary Patterns of Patients with Prediabetes and Type 2 Diabetes
Source: Metabolites. 2023 Apr 7;13(4):532. doi: 10.3390/metabo13040532 (PMC10142065; doi:10.3390/metabo13040532)
Supplement: Supplementary file 1 [file metabolites-13-00532-s001.zip › Table S5.pdf]

Table S5. Correlation analyses between dietary intake and lipid profile

	**Prediabetic**						**T2DM**					
	TG (mg/dL)		HDL (mg/dL)		LDL (mg/dL)		TG (mg/dL)		HDL (mg/dL)		LDL (mg/dL)	
	**r**	**p**	**r**	**p**	**r**	**p**	**r**	**p**	**r**	**p**	**r**	**p**
Meat and meat products (g)	0.028	0.650	-0.051	0.409	0.086	0.037	0.027	0.513	-0.063	0.130	0.052	0.205
Eggs and egg dishes (g)	0.010	0.875	-0.088	0.032	0.049	0.429	0.032	0.439	-0.006	0.881	0.004	0.926
Milk and milk products (g)	0.069	0.262	0.104	0.092	-0.029	0.645	-0.138	0.025	0.048	0.248	-0.052	0.205

TG, triglycerides; HDL, HDL cholesterol; LDL, LDL cholesterol.
